# Nuclear Accumulation of an Uncapped RNA Produced by Drosha Cleavage of a Transcript Encoding miR-10b and HOXD4

**DOI:** 10.1371/journal.pone.0025689

**Published:** 2011-10-03

**Authors:** Sze Lynn Calista Phua, V. Sivakamasundari, Yu Shao, Xiaohan Cai, Li-Feng Zhang, Thomas Lufkin, Mark Featherstone

**Affiliations:** 1 School of Biological Sciences, Nanyang Technological University, Singapore; 2 Genome Institute of Singapore, Singapore; Institute of Genetics and Molecular and Cellular Biology, France

## Abstract

Patterning of the animal embryo's antero-posterior (AP) axis is dependent on spatially and temporally regulated Hox gene expression. The murine *Hoxd4* gene has been proposed to harbour two promoters, an upstream promoter P2, and a downstream promoter P1, that lie 5.2 and 1.1 kilobase pairs (kb) upstream of the coding region respectively. The evolutionarily conserved *microRNA-10b* (*miR-10b*) gene lies in the *Hoxd4* genomic locus in the intron separating the non-coding exons 4 and 5 of the P2 transcript and directly adjacent to the proposed P1 promoter. *Hoxd4* transcription is regulated by a 3′ neural enhancer that harbours a retinoic acid response element (RARE). Here, we show that the expression profiles of *Hoxd4* and *miR-10b* transcripts during neural differentiation of mouse embryonal carcinoma (EC) P19 cells are co-ordinately regulated, suggesting that both *Hoxd4* and *miR-10b* expression is governed by the neural enhancer. Our observation that P1 transcripts are uncapped, together with the mapping of their 5′ ends, strongly suggests that they are generated by Drosha cleavage of P2 transcripts rather than by transcriptional initiation. This is supported by the colocalization of P1 and P2 transcripts to the same posterior expression domain in the mouse embryo. These uncapped P1 transcripts do not appear to possess an Internal Ribosomal Entry Site (IRES), but accumulate within multiple punctate bodies within the nucleus suggesting that they play a functional role. Finally, similar uncapped Drosha-cleaved P1-like transcripts originating from the paralogous *Hoxb4*/*miR-10a* locus were also identified. We propose that these transcripts may belong to a novel class of regulatory RNAs.

## Introduction

MicroRNAs are a class of highly conserved small noncoding RNAs (ncRNAs) expressed in a wide range of organisms [1], [2]. Like proteins, many miRNAs are encoded by genes transcribed by RNA Polymerase II to give a long primary microRNA (pri-miRNA) transcript which is 5′-capped and 3′-polyadenylated. The pri-miRNA forms a hairpin-loop structure that is cleaved at its base by an RNAse III enzyme, Drosha, to form the precursor microRNA (pre-miRNA) which is exported out of the nucleus and then cleaved again on the loop side of the hairpin by another RNAse III enzyme, Dicer. This generates an miRNA: miRNA* duplex, one strand of which is preferentially selected and incorporated into the RNA-induced silencing complex (RISC). The single stranded mature miRNA is typically 21–23 nucleotides long and functions by base-pairing to target complementary mRNAs to regulate gene expression. In animals, this regulation occurs mostly, but not always, at the post-transcriptional level [3]. Recent evidence suggests that miRNAs are also able to epigenetically silence genes at the transcriptional level [4].

Homeobox (Hox) genes encode homeodomain-containing transcription factors that control segmental patterning and determine the identity of embryonic regions along the AP axis before and during gastrulation in the mouse [5], [6]. They are highly conserved and found to be essential for normal development in all species where they have been tested [7]. Homeotic transformations and malformations in the embryo arise when Hox gene expression is deregulated by either a loss or gain of function, and the precise spatio-temporal control of their expression is therefore critical to normal development [6], [8], [9].

**Figure 1:**
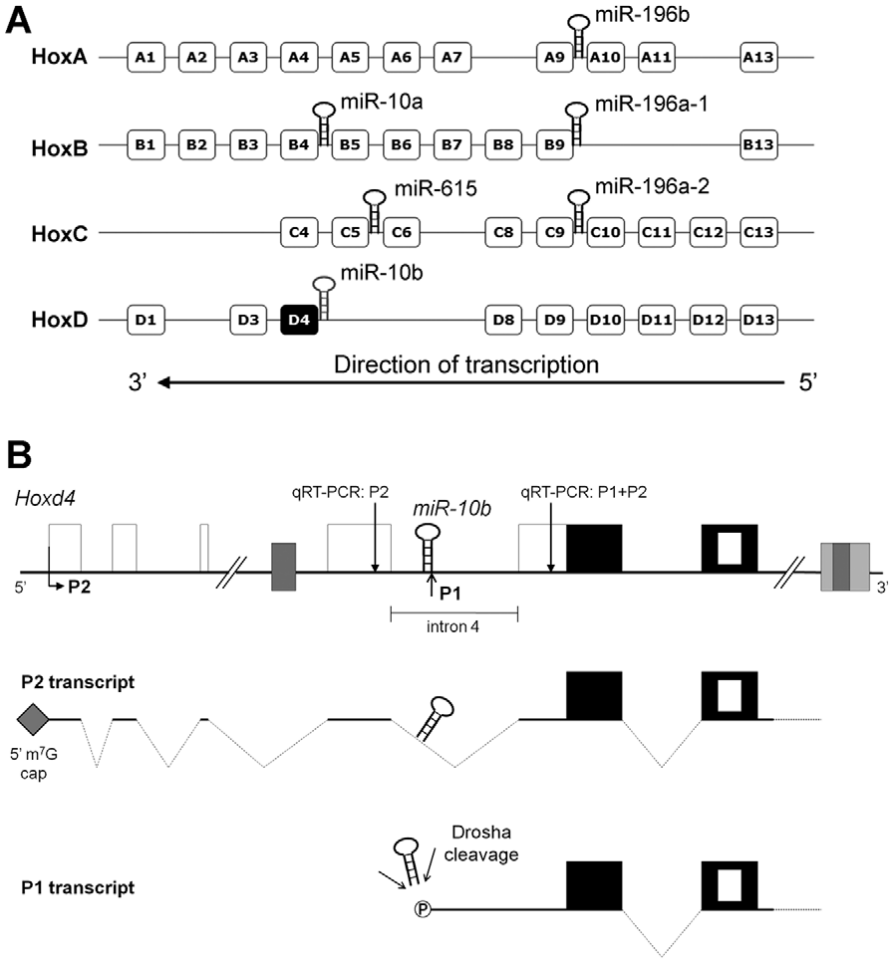
Hox genes and their associated microRNAs. (**A**) A schematic of the four mammalian Hox clusters (HoxA through HoxD) and the position of the microRNAs embedded within them [20]. The indicated direction of transcription applies to all four Hox clusters. (**B**) A schematic showing the genomic organization of the mouse *Hoxd4* gene. Empty boxes indicate non-coding exons and black boxes indicate coding exons. The nested white box indicates the homeobox within the second coding exon. Grey boxes show regulatory elements. P2 denotes the upstream promoter and P1 denotes the putative downstream promoter. *miR-10b* is found directly upstream of the P1 promoter, in intron 4 of the P2 transcript. Dotted lines indicate spliced introns. The grey diamond at the 5′ end of the P2 transcript denotes the 5′ 7-methylguanosine (m^7^G) cap and the circled “P” at the 5′ end of the P1 transcript indicates a 5′ phosphate. The positions of two 200 bp regions amplified by qRT-PCR (qRT-PCR∶P2 and qRT-PCR∶P1+P2) are indicated by vertical arrows (see figure 2).

In mammals, there are 39 Hox genes organized in 4 paralogous *Hox* clusters, A–D (Fig 1A). There are three known microRNAs or miRNA families embedded in vertebrate *Hox* clusters: *miR-10*, *miR-615* and *miR-196* (Fig. 1A). The position of these miRNAs within the *Hox* clusters is highly conserved during evolution. For example, both the position and sequence of the *miR-10* family are conserved in *Drosophila*, ancestral vertebrates, teleosts and mammals [10]–[12]. The *Hoxb4* and *Hoxd4* paralogs in mammals are orthologous to the *Deformed* Hox gene of flies. A *miR-10* family member is embedded 5′ to the coding region of each of these Hox genes. In mammals, the sequence of mature *miR-10a* and *miR-10b* differs by a single nucleotide. *miR-10a*, located upstream of *Hoxb4*, was reported to repress *Hoxd4* transcription by targeting its promoter region in human breast cancer cells [13]. *miR-10b* is found 5′ to *Hoxd4* and regulates metastasis and cell migration in human breast cancer cells *via* suppression of *Hoxd10* [14]–[16]. It is also known to be upregulated in malignant gliomas [17], pancreatic cancer [18] and chronic lymphocytic leukemia [19].

**Figure 2:**
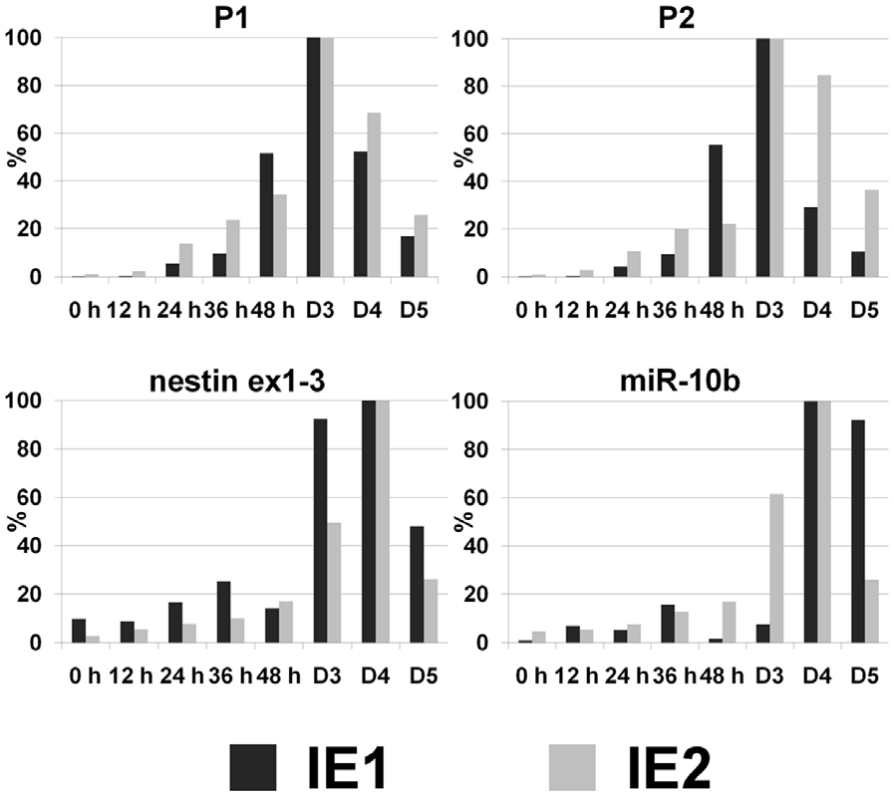
Expression profiles of *Hoxd4 * P1, P2 and *miR-10b* in differentiating P19 cells. qRT-PCR was carried out on cDNA derived from P19 cells undergoing neural differentiation. The levels of the transcripts were arbitrarily assigned 100% at the time point where they peaked. Transcripts of *nestin*, a marker of neural differentiation, accumulated with time during P19 differentiation, peaking by day 4 (D4). *miR-10b* expression was induced together with *Hoxd4* P1 and P2 transcripts and likewise peaked on day 4 (D4). P1, P2 and nestin were normalized to 18S RNA*; miR-10b* was normalized to U6 snoRNA. For specific detection of P2 transcripts only, or P1 plus P2 transcripts, primers were designed to amplify regions of approximately 200 bp whose positions are shown in figure 1B. Data represent two independent experiments, each shown as the average of qPCR technical duplicates. Dark grey bars: independent experiment 1 (IE 1); light grey bars: independent experiment 2 (IE 2).

*miR-10a* and *miR-10b* are expressed in the central nervous system and trunk in a sub-domain of the *Hoxb4* and *Hoxd4* expression domains. This spatio-temporal restriction along the AP axis is reminiscent of Hox gene expression and is conserved in many animals such as *Drosophila*, zebrafish and mouse [20]–[23].

*Hoxd4* patterns the anterior cervical skeleton of the mouse, and has been implicated in acute lymphoblastic leukemia [24]–[27]. Two *Hoxd4* promoters, an upstream promoter termed P2 and a downstream promoter termed P1 (Fig. 1B) have been deduced from the results of S1 nuclease and RNase protection assays, and 5′ Rapid Amplification of cDNA Ends (5′RACE) [26]. While these are rigorous methods to map the 5′ ends of RNA transcripts, they do not reveal whether these 5′ ends are capped.

In the mouse, the full *Hoxd4* expression domain in the developing central nervous system has an anterior limit at the boundary between rhombomeres 6 and 7 (r6/7) of the embryonic hindbrain. A region termed the 3′ neural enhancer (3′ NE) is essential for r6/7-restricted *Hoxd4* expression in the central nervous system of the developing mouse embryo as well as in differentiating P19 cells [28]–[34]. However, a subset of *Hoxd4* transcripts originating from an upstream promoter (P2) is expressed more posteriorly at a level just above the forelimb bud. *miR-10b* is expressed in a similar temporal and spatial pattern as the *Hoxd4* P2 transcripts in the developing E9.5 mouse embryo, with an anterior expression border that is considerably posterior to the r6/7 anterior expression border [23], [26]. The anterior expression border of *miR-10a* is likewise posterior to the r6/7 expression border of the full *Hoxb4* domain [20]. In zebrafish, *miR-10b* is expressed in the spinal cord with an anterior boundary somewhat posterior to the r6/7 boundary [35]. The zebrafish *miR-10* family is also found to repress *hoxb1a* and *hoxb3a* within the spinal cord in cooperation with *hoxb4a* [35].

The location of mouse *miR-10b* immediately adjacent to the presumptive P1 transcriptional start site and within the intron separating exons 4 and 5 of the P2 transcript (Fig. 1B) raised several questions regarding *Hoxd4* and the biogenesis of *miR-10b*. First, P2 may serve to encode both HOXD4 and *miR-10b*. In other words, the *Hoxd4* P2 transcript could also serve as the primary *miR-10b* transcript that is the substrate for processing by Drosha. Second, expression of the *Hoxd4/miR-10b* transcript may be dependent on the neural enhancer located 3′ to the *Hoxd4* coding region. Third, the previously mapped 5′ end of the *Hoxd4* P1 transcript may not be generated by transcriptional initiation but by the cleavage of the *Hoxd4* P2 transcript by Drosha (Fig. 1B).

Here, we show that these three hypotheses are correct. In addition, though P1 transcripts are not capped, they are abundant and largely localized to punctate sub-nuclear bodies.

## Results

### The expression of *Hoxd4* and *miR-10b* is co-ordinately regulated in differentiating P19 cells

To determine if the expression of pri-*miR-10b* is controlled by the *Hoxd4* neural enhancer, the relative levels of *Hoxd4* P1, P2 and *miR-10b* transcripts were measured in differentiating P19 mouse EC cells. P19 cells can be induced to undergo neuronal differentiation by treatment with all-*trans* retinoic acid (RA) and cell aggregation [36]. P19 neural differentiation recapitulates aspects of normal embryonic differentiation whereby Hox genes are expressed sequentially in a colinear manner [33], [37]–[40]. All *Hoxd4* P1, P2 and *miR-10b* transcripts were barely detectable in undifferentiated P19 cells (Fig 2, P1, P2, miR-10b at 0 h). The accumulation of *nestin* transcripts, a neural lineage marker, served as a marker for the P19 differentiation process. *Hoxd4* P1 and P2 transcripts were highly induced during P19 differentiation, peaking at maximum levels on day 3 of differentiation (Fig 2, P1, P2 at D3). In a fashion similar to the *Hoxd4* transcripts, the *miR-10b* transcripts were first expressed at very low levels in undifferentiated cells and were then strongly induced upon RA treatment and aggregation, peaking at day 4 (Fig 2, miR-10b at D4). When compared to the *Hoxd4* P1 and P2 transcripts, we observed that the *miR-10b* peaked a day later, on day 4. This delay in the expression peak may reflect differences in processing or stability; however, the overall expression profile is similar. This is in contrast to the expression profiles of other Hox genes such as *Hoxa1* which is induced by 6 h and peaks as early as 48 h of neural differentiation [37], [39].

In summary, *miR-10b* expression was shown to be induced together with *Hoxd4* P1 and P2 transcripts during P19 differentiation. These data are consistent with a role for the *Hoxd4* 3′ neural enhancer in directing expression of both *Hoxd4* and *miR-10b* transcripts.

### *Hoxd4* transcripts originating from P1 are generated by Drosha cleavage

To determine if *Hoxd4* P1 transcripts are generated by Drosha cleavage or transcriptional initiation, an RNA-ligase mediated 5′ Rapid Amplification of cDNA Ends (5′ RLM-RACE) was performed. Transcripts whose 5′ ends are generated by Drosha cleavage will bear a 5′ phosphate and will be suitable substrates for RNA ligase, unlike intact transcripts synthesized by RNA pol II which will have a 5′ 7-methylguanosine (5′ m^7^G) cap and therefore be unable to participate in RNA-ligase-mediated reactions.

**Figure 3:**
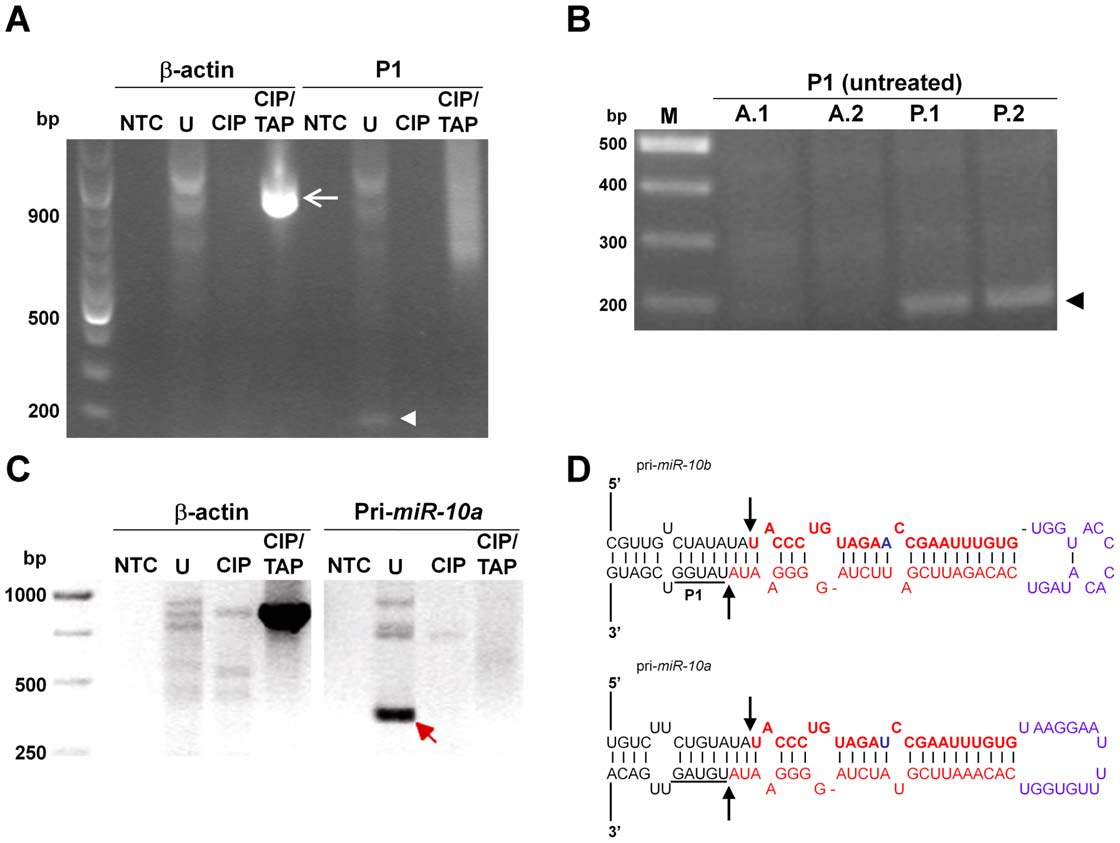
*Hoxd4* P1 transcripts are generated by Drosha cleavage. (**A**) Presence of uncapped P1 transcripts. The untreated sample (U) gave an approximately 188 bp amplification product (arrowhead). As a positive control, an expected 900 bp product from β-actin is amplified only when the RNA has been treated with CIP and then TAP (arrow). NTC: no template control; U: untreated RNA; CIP: CIP-treated RNA; CIP/TAP: CIP and TAP-treated RNA. (**B**) Uncapped P1 transcripts are present only in posterior tissues of the mouse embryo. Untreated (U) RNA from the posterior tissues of two different mouse embryos (P.1, P.2) gave a 188 bp amplification product by RT-PCR that corresponds to the presence of uncapped P1 transcripts (black arrowhead). This band is at background levels in anterior tissues of two mouse embryos (A.1, A.2) where *Hoxd4* is not expressed. M: molecular weight markers. (**C**) Presence of uncapped *Hoxb4* transcripts. The untreated sample (U) gave a 300 bp amplification product (red arrow), indicating the presence of an uncapped *Hoxb4* transcript. Image is the negative of the ethidium bromide stained gel. (**D**) Drosha cleavage site of pri-*miR-10b and -10a*. The nucleotides in red show the miRNA duplex formed by the mature *miR-10b/a* (top strand) and miR-10b/a* (bottom strand) sequence. Black arrows show the Drosha cleavage sites on the pri-*miR-10b/a* hairpin. The cleavage site is exactly 11 bp from the bottom of both the pri-*miR-10b* and pri-*miR-10a* stem junction on the downstream side. This is within a single nucleotide of the previously mapped P1 start site (a cluster of 4 nt underlined and denoted “P1” on pri-*miR-10b*).

Total RNA from P19 cells on day 3 of differentiation was collected. An aliquot was treated with calf intestinal phosphatase (CIP) to remove all free 5′ phosphates in the total RNA. An aliquot of the CIP-treated RNA was subsequently treated with tobacco acid pyrophosphatase (TAP), which removes the 5′ cap on mRNA transcripts to leave a free 5′ phosphate. This was followed by ligation of all three RNA samples (untreated, CIP-treated and CIP/TAP-treated) to a synthetic RNA oligonucleotide and RT-PCR. Only transcripts bearing a 5′ phosphate, such as produced by Drosha cleavage (Fig 1B), can be amplified from untreated RNA samples. The untreated RNA sample gave a band of approximately 190 bp after PCR, which corresponded to the 188 bp from the primer to the predicted Drosha cleavage site (Fig 3A, arrowhead). Moreover, no comparably sized products were amplified from the CIP/TAP-treated RNA which would have allowed detection of capped transcripts (Fig 3A). The mRNA for a β-actin control was amplified only following treatment with both CIP and TAP (Fig 3A, arrow), as expected for a capped transcript.

Next, we confirmed the presence of these presumptive Drosha-cleaved P1 transcripts in the mouse embryo. The otic vesicle is a morphological landmark that lies just anterior to the *Hoxd4* expression border at r6/7. Embryonic day (E) E9.5 mouse embryos were therefore bisected just anterior to the developing otic vesicle and RNA extracted from anterior (*Hoxd4*-negative) and posterior (*Hoxd4*-expressing) tissues followed by 5′ RLM-RACE. The 188 bp band indicating the presence of Drosha-cleaved P1 transcripts was amplified only from posterior tissue (Fig 3B). This demonstrates that the presumptive Drosha-cleaved P1 transcripts are generated during normal mouse embryonic development and only in embryonic regions expressing *Hoxd4*.

The approximately 188 bp band derived from P19 cell RNA was then cloned and sequenced. The 5′ ends of all four clones began with 5′-TATGG-3′, mapping precisely to the predicted Drosha cleavage site on the pri-*miR-10b* transcript, 11 nt from the base of the pri-miRNA stem junction [41]. Importantly, this site is within one nucleotide of the previously deduced P1 start site cluster. Together, these observations suggest that P1 transcripts are generated by Drosha cleavage of the primary microRNA for *miR-10b*. The most likely origin for pri-*miR-10b* is a transcript initiating at P2 (Fig. 1B). In other words, pri-*miR-10b* and the *Hoxd4* P2 transcript are one and the same.

### Drosha cleavage of the *Hoxb4*/*pri-miR-10a* transcript generates similar uncapped *Hoxb4* transcripts

Similar to *miR-10b* and *Hoxd4*, *miR-10a* is located in a conserved position 5′ to *Hoxb4* (Fig. 1A). qPCR analysis showed that both *miR-10a* and *Hoxb4* transcripts are induced in a similar manner to *miR-10b* and *Hoxd4* transcripts during RA-induced P19 neural differentiation (unpublished observations). To determine if there was a Drosha-cleaved *Hoxb4*/pri-*miR-10a* transcript paralogous to *Hoxd4* P1, we carried out 5′-RLM-RACE with a *Hoxb4*-specific reverse primer (RACE-Hoxb4-R). Only the untreated RNA sample gave an amplification product of 300 bp corresponding to the distance between the reverse primer and the predicted Drosha cleavage site (Fig 3C, arrow). Sequencing of the *Hoxb4* PCR product confirmed that it is indeed *Hoxb4*-specific, mapping it to the predicted Drosha cleavage site precisely 11 nt from the base of the pri-miRNA stem junction, just as for *Hoxd4* (Fig 3D). This indicated the presence of similar uncapped Drosha-cleaved *Hoxb4* transcripts in differentiating P19 cells, suggesting that they may belong to a novel class of RNA species.

### Drosha-cleaved *Hoxd4* P1 and P2/pri-*miR-10b* transcripts are all expressed posterior to the rhombomere 6/7 boundary in the mouse embryo

**Figure 4:**
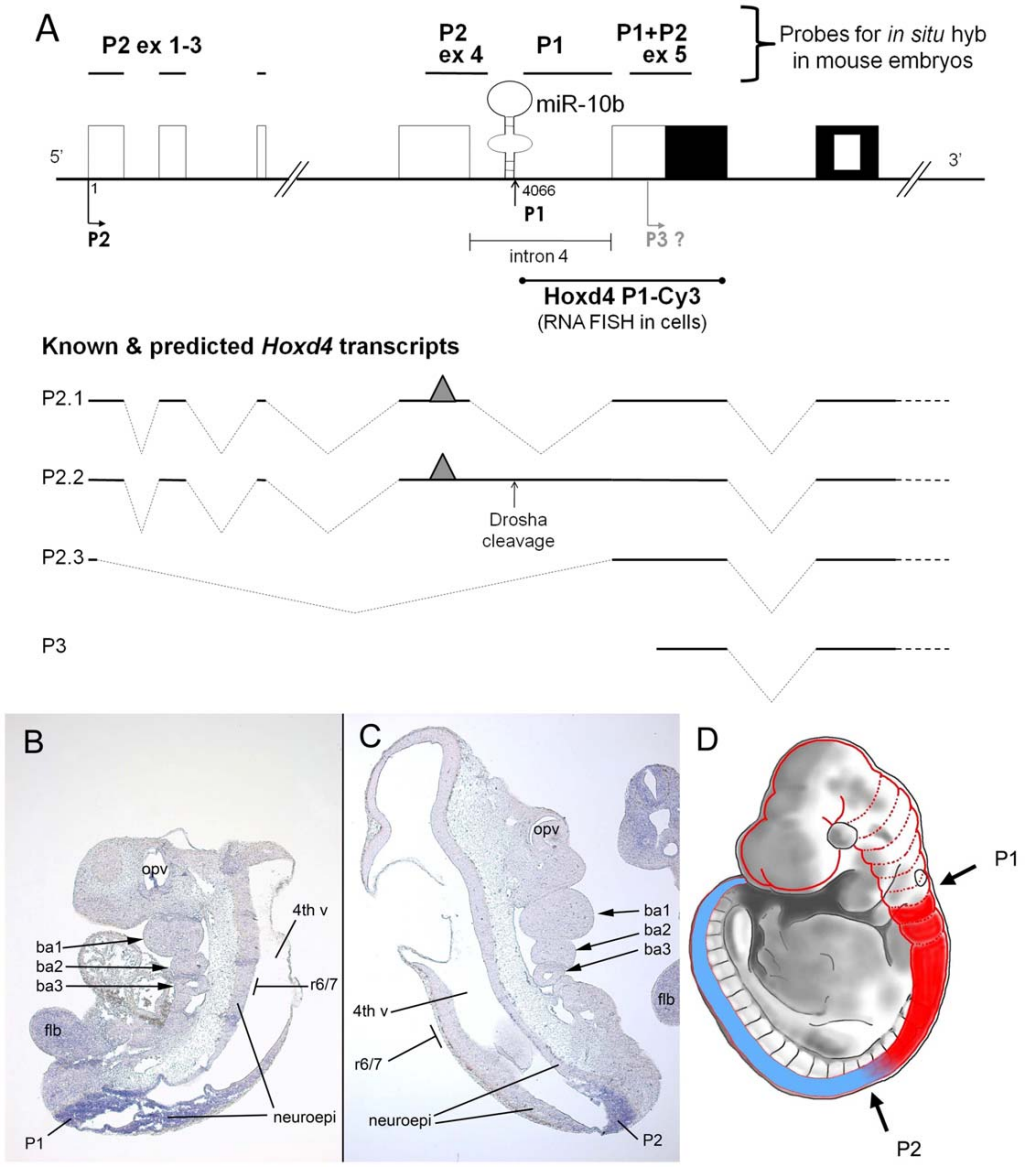
Distribution of *Hoxd4/miR-10b* transcripts along the embryonic AP axis. (**A**) *Hoxd4* probes and transcripts. The *in situ* hybridization probes are shown above a diagram of the *Hoxd4* genomic locus. Previously, the P2 ex 4 probe spanning exon 4 and extending partially into intron 4, detected P2 transcripts at a point posterior to the r6/7 boundary, while the P1+P2 ex 5 probe spanning exon 5 detected P1+P2 transcripts with an r6/7 boundary. The newly designed P2 ex 1-3 probe spanned the first 3 exons, and detected spliced P2 transcripts, while the P1 probe spans the 5′ UTR of P1 transcripts, a region that is spliced out in mature P2 transcripts. Exons are denoted as for Fig. 1B. The P1 5′ end maps to nucleotide 4066 downstream of the major P2 start site. The *Hoxd4* P1-Cy3 probe used for RNA FISH in Fig 5D and E extends from the P1 5′ end to the 3′ end of *Hoxd4* exon 5 as drawn. The grey triangle represents a hypothetical mRNA destabilizing element that is proposed to be absent from hypothetical transcript P2.3. (**B,C**) Expression of *Hoxd4* in E9.5 mouse embryos. Both the Drosha-cleaved P1 transcript detected by the P1 probe (**B**) and the P2 transcript detected by the P2 ex 1-3 probe (**C**) show anterior expression boundaries that are posterior to the anterior limit of the full *Hoxd4* expression domain at r6/7. ba1,2,3: branchial arches 1, 2 and 3; 4^th^ v: fourth ventricle within the developing brain; neuroepi: neuroepithelium; flb: forelimb bud; opv: optic vesicle. The r6/7 boundary is approximately opposite branchial arch 3 as indicated. (**D**) Schematic of distribution of *Hoxd4* transcripts in the E9.5 mouse embryo. The solid red plus blue shading represents the cumulative distribution of all *Hoxd4* transcripts. The most anteriorly expressed transcripts (red) have an anterior expression border at the r6/7 boundary. Such an expression pattern is detected with the P1+P2 ex 5 probe [26] as well as probes spanning the homeobox-containing exon and 3′ UTR [34], [40], [45]. The region in blue represents the expression domain of those *Hoxd4* transcripts detected by the more upstream probes, P2 ex 1-3, P2 ex 4 and P1 [26].

Using a probe against the *Hoxd4* 5′ coding and non-coding region (P1+P2 ex5, Fig. 4A), *Hoxd4* transcripts have been shown to have an anterior expression boundary between r6 and r7 within the developing hindbrain of mice. This expression border is conserved in zebrafish [23], [26]. By contrast, a probe that specifically detected P2 transcripts (probe name  =  P2 ex4, Fig. 4A) revealed a more posterior expression boundary in the anterior spinal cord above the forelimb bud [26]. Thus, the anterior part of the *Hoxd4* expression domain up to r6/7 was attributed to the activity of a distinct P1 promoter. However, given our observation that P1 transcripts are likely generated by Drosha cleavage of P2 transcripts, and therefore do not originate from a transcriptional start site, we re-visited the spatial distribution of P1 and P2 transcripts in the mouse embryo.

**Figure 5:**
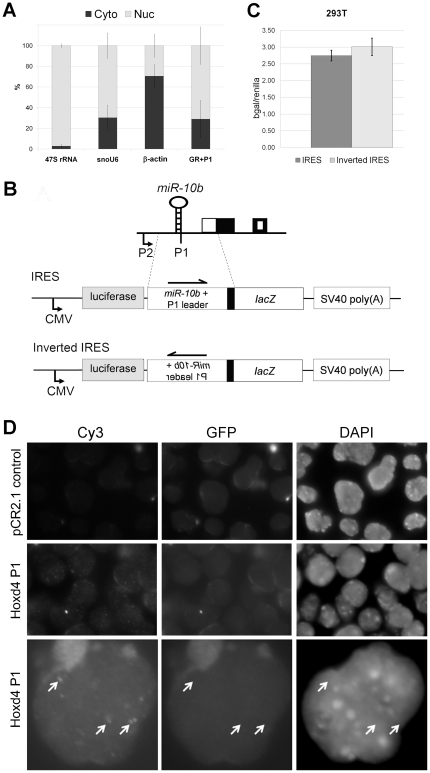
Drosha-cleaved P1 transcripts are not translated and are localized to punctate nuclear bodies. (**A**) qRT-PCR of transcripts in nuclear (Nuc, light grey bars) and cytoplasmic (Cyto, dark grey bars) fractions of P19 cells on day 3 of differentiation. 47S pre-rRNA and snoU6 RNA served as positive controls which were enriched in the nuclear fraction while β-actin mRNA was more abundant in the cytoplasmic fraction. Almost three-quarters of the total Drosha-cleaved P1 transcripts (white) detected were present in the nuclear fraction. Data shown represent the mean of experimental triplicates, and error bars are plotted as the percentage of the standard deviation to mean. (**B**) IRES reporter assay in 293T cells. The activity of the IRES construct is not significantly higher than the control inverted IRES construct in either 293T cells. β-gal activity was first normalized to renilla luciferase activity. Data shown represent the mean of experimental triplicates, and error bars give standard deviation. (**C**) Schematic of the IRES and control reporter constructs. The IRES test construct consists of the luciferase cassette driven by a CMV promoter, followed by the P1 putative IRES region in front of the *lacZ* gene and SV40 polyadenylation signal. The inverted IRES construct consists of the same components except that the putative IRES region, which is about 1 kb in length, is inverted. Boxes indicate exons as per figure 1B. (**D**) RNA FISH of P19 cells on day 3 of neural differentiation. Vertical columns show images obtained under excitation for Cy3, GFP and DAPI. Images obtained with excitation for GFP control for autofluorescence. The upper row shows a negative control Cy3-labelled probe (pCR2.1 control) and the middle and lower rows show the localization of the Cy3-labelled *Hoxd4* P1 probe (Hoxd4 P1). *Hoxd4* transcripts are specifically detected as speckles in the nucleus of neurally differentiated P19 cells. Images in the bottom row have been digitally enlarged in order to present the punctate bodies more clearly. The Adobe Photoshop application was used to enhance the contrast *simultaneously* to pairs of images in the Cy3 and GFP columns. Arrows denote punctate bodies visible with the Cy3-labelled Hoxd4 P1 probe. These bodies are not visible when the sample is excited for GFP and do not correspond to chromatin structures detected by DAPI.

We designed two new *in situ* hybridization probes to more precisely investigate the expression of these different transcripts in the embryo. First, a P1 probe was designed to be just downstream of *miR-10b* and spanning the 5′ untranslated region (5′ UTR) of the Drosha-cleaved P1 transcripts (probe name  =  P1, Fig. 4A). This region is spliced out in the P2 transcripts and thus the P1 probe will specifically detect P1 transcripts only. A second probe (probe name  =  P2 ex 1–3, Fig. 4A), spans the first 3 exons of the P2 transcript and detects P2 transcripts only. *In situ* hybridizations with both the P1 and P2 ex 1–3 probes showed an expression domain that is posterior to the r6/7 boundary, similar to the expression domain of mature *miR-10b* in the E9.5 embryo. Neither probe detected more anterior expression up to the r6/7 boundary (Fig 4B, C, D). We conclude that P1 transcripts are derived by Drosha-cleavage of transcripts originating at the posteriorly active P2 promoter. The more anterior expression (up to r6/7) previously detected with probe P1+P2 ex 5 may be due to the action of a more anteriorly active promoter (hypothetical transcript P3, Fig. 4A). Alternatively, P2 may be the only *Hoxd4* promoter, but the presence or absence of destabilizing elements due to differential splicing determines whether P2-derived transcripts accumulate at anterior or posterior positions (hypothetical transcript P2.3, Fig. 4A).

### Drosha-cleaved P1 transcripts are abundant in the nucleus

We determined that P1 transcripts are generated by Drosha cleavage. However, these uncapped P1 transcripts appeared to be abundant in differentiating P19 cells (Fig 2) and the mouse embryo (Fig 3B) [26]. We therefore asked if these abundant, uncapped P1 transcripts may have a function. As a first indication, we determined whether the uncapped P1 transcripts were present in the cytoplasm or nucleus.

Nuclear-cytoplasmic fractionation of neurally differentiating P19 cells was performed, followed by RLM-PCR as described above. The controls – 47S pre-rRNA and snoU6 RNA – were correctly enriched in the nuclear fraction while β-actin mRNA was more abundant in the cytoplasmic fraction as expected (Fig 5A). We found almost three quarters of *Hoxd4* P1 transcripts in the nuclear fraction.

### Absence of robust IRES activity within the 5′ UTR of P1 transcripts

The presence of approximately one quarter of P1 transcripts in the cytoplasm suggested that they may be translated *via* an IRES. To determine whether the 5′ UTR of the cleaved P1 transcript has IRES activity, an IRES reporter assay was designed. The IRES reporter consisted of a firefly luciferase cassette driven by a CMV promoter, followed by the putative IRES region driving *lacZ* gene expression and ending with an SV40 polyadenylation signal. The “Inverted IRES” construct had the putative IRES region inverted and cloned in the same position within the vector and served as a negative baseline control (Fig 5B). Any increase in β-galactosidase levels of the IRES construct compared to the Inverted IRES construct would thus reflect IRES activity.

The IRES test plasmids were co-transfected with control renilla luciferase expression vectors in either HEK293T cells or RA-treated P19 cells. Cell lysates were assayed at 48 h post-transfection and β-galactosidase was normalized to renilla luciferase activity to account for variations in transfection efficiency. No significant IRES activity was observed in the HEK293T cells (Fig 5C). There was also no significant β-galactosidase activity in transfected and RA-treated P19 cells compared to untransfected cells (data not shown). This suggests that the Drosha-cleaved P1 transcripts are not translated through an IRES. This result also indicates the absence of any additional promoter activity in the putative IRES region that stretches for about 1 kilobase pairs upstream from the *Hoxd4* coding region. Efficient cleavage of the IRES reporter fusion transcript by Drosha was evident from the fact that firefly luciferase activity derived from this reporter was low by comparison to that obtained with the Inverted IRES reporter (data not shown).

### *Hoxd4* P1 transcripts localize to punctate nuclear bodies

We performed RNA fluorescence *in situ* hybridization (RNA FISH) to determine sub-localization of nascent *Hoxd4* and P1 transcripts in day 3 differentiated P19 cells. A Cy3-labelled *Hoxd4* P1 probe spanning 1.5 kb extending from the Drosha cleavage site to the *Hoxd4* coding region (Fig 4A) yielded multiple signals corresponding to punctate nuclear bodies (Fig 5D, middle and bottom rows). A negative control Cy3-labelled probe (pCR2.1 control) yielded no specific signal (Fig 5D, top row). Likewise, undifferentiated P19 cells which do not express *Hoxd4* did not yield any signal (data not shown). Thus, endogenous *Hoxd4* P1 transcripts are abundant in the nucleus and accumulate in a pattern suggestive of function.

## Discussion

We have established that the 5′ ends of *Hoxd4* P1 transcripts are not capped, bear a terminal phosphate and map to the predicted Drosha cleavage site at the base of the stem of the pri-*miR-10b* stem-loop. The 5′ end of the *Hoxd4* P1 transcript is precisely 11 nucleotides from the base of the pri-miRNA stem junction. A similar result was obtained for the Drosha cleavage product of *miR-10a* and *Hoxb4*. This result is consistent with a validated model in which Drosha cleaves at a position 11 nt from the stem-ssRNA junction [41]. We conclude that *Hoxd4* P1 transcripts are indeed the result of Drosha cleavage of the pri-*mir-10b* transcript and, contrary to our previous interpretation [26], are not generated by transcriptional initiation from a distinct promoter, but by the action of Drosha on transcripts initiated at the P2 promoter.

### A shared promoter and common regulatory elements for *Hoxd4* and *miR-10b*

A significant majority of human miRNAs resides in intronic regions and in the same orientation as the host coding genes [42]. The expression patterns of these intronic miRNAs frequently coincide with the genes in which they are embedded, indicating that they could be regulated by common *cis*-regulatory elements [43]. Expression of *Hoxd4* transgenes in P19 cells and in transgenic mouse embryos is critically dependent on a 3′ neural enhancer [28], [31]–[34].**** The expression profiles of *Hoxd4* and *miR-10b* during P19 differentiation as measured by qRT-PCR are similar, with low basal expression levels in undifferentiated P19 cells and strong induction of their expression upon neural differentiation, peaking at day 3 or 4 and declining thereafter. This is in contrast to the expression profiles of other Hox genes in P19 cells such as *Hoxa1* which has been shown to be induced as early as 6 hours of neural differentiation [37]. Our data are therefore consistent with both *Hoxd4* and *miR-10b* transcripts coming under the control of this same regulatory region.

Primary miRNAs transcribed by RNA polymerase II are 5′ capped and 3′ polyadenylated, making them structurally identical to messenger RNAs. It has been reported that human pri-miRNA transcripts also give rise to mRNA coding for a protein [44]. This is consistent with our observation that transcripts initiating at *Hoxd4* P2 have the potential to encode both *miR-10b* and HOXD4. Such an arrangement would facilitate the co-regulated expression of both *Hoxd4* and *miR-10b* during development, as activation of the P2 promoter would lead to production of both *miR-10b* and HOXD4. This is supported by *in situ* data that showed an extensive overlap of mature *miR-10b* and *Hoxd4* expression both spatially and temporally [23], [26]. The coordinated regulation of both genes suggests that they may have shared functions during early development such as has been described for the shared repressive functions of the miR-10 family and *hoxb4* in zebrafish [35].

### Expression along the antero-posterior axis

The full *Hoxd4* expression domain extends anteriorly to the boundary between r6 and r7 in the developing hindbrain (Fig. 4D, combined blue plus red shading) as revealed by *in situ* probes overlapping the 5′ or 3′ ends of the coding region [26], [34], [40], [45]. However, *miR-10b* and *Hoxd4* P2 and P1 transcripts are not detected in the anterior-most *Hoxd4* expression domain up to r6/7 [26]. If a single P2 promoter drives expression of all transcripts derived from the *Hoxd4* locus, then there must be post-transcriptional controls which prevent some RNAs from accumulating in anterior tissues up to r6/7. In one possible mechanism, P2 transcripts that include a hypothetical destabilizing element (grey triangle in Fig. 4A) located in the *Hoxd4* 5′ UTR are unstable and degraded in the anterior-most part of the *Hoxd4* expression domain up to r6/7 (Fig 4D, shaded red). In this scenario, only alternatively spliced transcripts that lack this destabilizing element, such as the hypothetical P2.3 transcript shown in figure 4A, accumulate in the anterior-most domain.

Alternatively, an as-yet uncharacterized promoter (hypothetical promoter P3, Fig. 4A) is active in anterior neural tissue and is responsible for expression in the anterior-most portion of the *Hoxd4* expression domain. This is supported by the presence of cDNA and Expressed Sequence Tag (EST) clones (FANTOM) whose 5′ ends map immediately upstream of the *Hoxd4* coding region. In addition, on the basis of primer extension results, a putative human *HOXD4* promoter has been mapped 21 bp 5′ of the ATG start codon [46]. However, such *Hoxd4* transcripts may not be expressed in the neural lineage, and we have been unable to detect *Hoxd4* 5′ ends in the P3 region despite extensive S1 nuclease and RNase protection assays [26] and 5′RLM-RACE (unpublished data). In addition, the results presented in figure 5C fail to reveal promoter activity in the 1 kb sequence upstream of the *Hoxd4* ATG start codon. The lack of a promoter at presumptive P3 is further substantiated by a low density of elongating RNA pol II, p300, TBP and H3K4me3 adjacent to the *Hoxd4* coding region. Supporting one or more far upstream promoters, the density of these four factors is high in a broad region spanning P2 (ChIP-seq data from CH12 cells from ENCODE/Stanford/Yale displayed on the UCSC Genome Browser)[47].

The relationship between the *miR-10* family and *Hox4* genes is surprisingly well conserved through evolution. In the zebrafish genome, three *miR-10* members, *miR-10b-1*, *miR-10b-2*, and *miR-10c*, are positioned upstream of the Hox group 4 paralogs *hoxd4a*, *hoxc4a* and *hoxb4a*, respectively [48]. The remaining two miR-10 family members, *miR-10c* and *miR-10d*, are located at homologous positions near sites from which 4^th^ group paralogs have been lost in the *HoxBa* and (vestigial) *HoxDb* clusters [48]. The sequences of mature *miR-10b-1* and *miR-10b-2* are identical and they are expressed slightly posterior to the r6/7 boundary, reminiscent of the situation in the mouse embryo [23], [26], [35].

Long intergenic primary Hox transcripts have been documented from the earliest days of the field [49]. More recently, high-resolution transcriptional profiling of the Hox clusters has revealed extensive polycistronic transcripts and a high degree of transcriptional complexity within the mammalian Hox clusters [50]. This increases the possible sources of transcripts acting as primary microRNAs and/or Hox messenger RNAs. A long primary transcript (Genbank BK005082) originating downstream of zebrafish *hoxb5a* spans *miR-10c, hoxb4a* and *hoxb3a* and is expressed in a domain posterior to *hoxb4a* [35], [51]. A similar situation exists in mouse where a long-range *Hoxd3* transcript initiated from the *Hoxd4* P2 promoter has the potential to code for *miR-10b* as well (NM_010468). Thus, it may be generally true of Hox complexes that long intergenic transcripts governed by a variety of enhancers contribute to miRNA accumulation at different points along the antero-posterior axis.

### Putative function of Drosha-cleaved P1 transcripts

Although uncapped transcripts are typically degraded rapidly [52], the *Hoxd4* P1 product of Drosha cleavage appears to be both stable and abundant in the mouse embryo and neurally differentiating P19 cells. We have also shown the presence of uncapped Drosha-cleaved *Hoxb4* transcripts (Fig 3C), leading us to speculate that this may be a new class of RNA species with novel functions.

While P1 transcripts might be translated, they do not appear to have an IRES necessary for cap-independent translation (Fig 5C). By contrast, however, P1 transcripts are abundantly localized to punctate nuclear bodies in differentiating P19 cells. There is now widespread evidence for the prevalence and importance of actively transcribed ncRNAs in mammalian cells [53]–[55]. The highly regulated expression of these ncRNAs in a temporal or spatial manner is suggestive of functional significance. Some Hox-associated ncRNAs which are 5′ capped, spliced and polyadenylated, such as HOTAIR and HOTTIP were found to interact with Polycomb/PRC2 and Trithorax/MLL complexes, respectively, controlling histone methylation and chromatin remodeling and thereby silencing or activating transcription [56], [57]. Intriguingly, the HOTAIR ncRNA employs a *trans* mode of action by silencing transcription at a distant HOXD locus while the HOTTIP ncRNA activates gene expression in *cis*, on the proximal HOXA genes. It is thus conceivable that the *Hoxd4* P1 transcripts may perform similar roles by binding chromatin remodeling complexes to direct transcription of genes in *trans* or *cis*. Further insight will require the use of appropriate probes to co-localize P1 transcripts to one of the known sub-nuclear structures such as paraspeckles [58].

## Materials and Methods

### Ethics statement

All animal procedures were performed according to the Singapore A*STAR Biopolis Biological Resource Center (BRC) Institutional Animal Care and Use Committee (IACUC) guidelines and the IACUC protocols employed were reviewed and approved by the aforementioned committee before any animal procedures were undertaken for this study described here (IACUC Protocol No: 080348 and 080377).

### Cell culture and differentiation

P19 cells (American Type Culture Collection #CRL-1825) were cultured in DMEM (Gibco) supplemented with 10% fetal bovine serum. For differentiation, the P19 cells were seeded at a density of 1×10^5^ cells/ml in a 10 cm untreated polystyrene bacterial petri dish (Greiner Bio-One, Germany), treated with 0.3 µM of all-trans retinoic acid and allowed to aggregate. This was designated as day 0. Fresh media with 0.3 µM RA was replaced after 2 days. After 4 days, fresh media without RA was replaced and the cells plated onto tissue culture plates (Corning, USA).

### Quantitative reverse transcriptase PCR

RNA was extracted using the PureLink™ Micro-to-Midi™ Total RNA Purification System (Invitrogen). 1 µg total RNA was first treated with 1 U DNase I (Fermentas) at 37 °C for 30 min. Reverse transcription of mRNA was carried out with SuperScript® III First-Strand Synthesis (Invitrogen) and reverse transcription of miRNA was carried out with NCode™ miRNA First-Strand cDNA Synthesis (Invitrogen) as per the manufacturer's instructions. The cDNA was then used for quantitative PCR (qPCR) together with SYBR GreenER^™^ qPCR SuperMix (Invitrogen) on a BioRad iCycler iQ5. 18S RNA was used as internal control for the mRNAs while U6 RNA was used as an internal control for the microRNAs.

### RNA ligation mediated 5′ rapid amplification of cDNA ends

RNA was extracted from P19 cells on day 3 of differentiation using the PureLink™ Micro-to-Midi™ Total RNA Purification System (Invitrogen) and 5′ RACE performed as per manufacturer's instructions (GeneRacer® Kit, Invitrogen).

### RNA *in situ* hybridization

Embryos were fixed overnight at 4°C in 4% paraformaldehyde and subsequently processed for 10 µm paraffin-embedded sections as described [59]. RNA *in situ* hybridization with digoxigenin (DIG)-labeled probes was performed as previously described [60]. The following plasmids were used as templates for synthesizing antisense DIG-labeled RNA probes: pGEMT Hoxd4 uP1 and pCR4-TOPO Hoxd4 P2 exon1-3. Following hybridization and washing, sections were stained with NBT/BCIP and exposed overnight at 4°C in dark according to manufacturer's instructions (Roche). Sections were subsequently washed in PBS and mounted with glycerol gelatin. All sections were photographed using a Zeiss Axio Imager Z1.

### RNA FISH

Day 3 neurally differentiated P19 cells were typsinized and diluted to a concentration of 7×10^5^ cells/ml before being cytospun onto glass slides using a cytocentrifuge, CytoSpin 4 (Thermo Scientific). Cells were then washed with ice-cold PBS for 5 min followed by fixation at room temperature in 4% paraformaldehyde (PFA) for 10 min. The slides were stored in 70% ethanol at 4°C. Before use, the slides were sequentially dehydrated through 80, 90 and 100% ethanol for 2 min each. The RNA FISH probes were labelled with the Nick translation kit and Cy3-dUTP according to the manufacturer's instructions (Roche). Cot-1 mouse DNA was added to the Cy3-labelled probe and stored in hybridization buffer (50% formamide, 2x SSC pH 7.4, 2 mg/ml BSA, 10% Dextran Sulfate-500K) with a final concentration of 500 ng/µl and 50 ng/µl respectively. Hybridization was carried out at 42°C for 3 hours, followed by three washes of 5 min each in 50% (v/v) formamide in 2x SSC and another three washes of 5 min each in 2x SSC at 45°C. All cells were imaged using a Nikon Eclipse T*i*.

### Nuclear-cytoplasmic fractionation

Day 3 neurally differentiated P19 cells (∼10^6^ cells) were pelleted and washed twice in PBS. Cells were then resuspended in 0.5 ml chilled lysis buffer (10 mM Tris pH 7.4, 3 mM MgCl_2_, 10 mM NaCl, 150 mM sucrose, 0.5% NP-40) with 10-20 U of RNaseOUT (Invitrogen) and kept on ice for 5–10 min with gentle mixing every minute. The cell lysate was centrifuged at 250 g in a microcentrifuge at 4°C for 5 min. Supernatant containing the cytoplasmic fraction was collected. The nuclear pellet was washed twice with 1 ml lysis buffer without NP-40 and resuspended in 100 µl of lysis buffer. RNA extraction of both nuclear and cytoplasmic fractions was performed with RNA mini kit (Invitrogen) followed by phenol-chloroform (pH 4.7) extraction and ethanol precipitation. RNA was then resuspended in equal volumes of DEPC-treated water. Equal volumes (1–2 µl) of the total RNA obtained from the nuclear and cytoplasmic fractions was then individually ligated to the RNA oligonucleotide adaptor using T4 RNA ligase (Fermentas). Reverse transcription of mRNA was carried out with SuperScript® III First-Strand Synthesis (Invitrogen). The cDNA was then used for qPCR together with SYBR GreenER^™^ qPCR SuperMix (Invitrogen) on a BioRad iCycler iQ5.

### IRES plasmid transfection

293T cells (American Type Culture Collection #CRL-11268) were seeded at a density of 1×10^5^ cells/ml in a 12-well plate and allowed to attach overnight. Transfection was performed the next day with Lipofectamine 2000 (Invitrogen) and 1.2 µg of IRES or inverted IRES plasmids together with 50 ng of renilla reporter plasmid. Cell lysate was extracted 2 days after transfection. β-gal activity was measured using the Dual-Light® Combined Reporter Gene Assay System (Applied Biosystems) and normalized to renilla activity which was measured using the Dual**-**Luciferase™ Reporter Assay System (Promega) on a Fluoroskan Ascent FL (Thermo). Experimental triplicates were performed.

### Plasmid constructs

The pGEMT *Hoxd4* uP1 plasmid used for mouse *in situ* hybridizations was made by T-tailed cloning of an approximately 700 bp PCR fragment amplified from pSNlacZpA (Zhang *et al.* 2000) starting from the P1 start site to the 3′ splice acceptor of *Hoxd4* intron 4 into the pGEMT vector (Promega).

The pCR4-TOPO *Hoxd4* P2 exon 1-3 plasmid used for mouse *in situ* hybridizations was made by T-tailed cloning of an 287 bp PCR fragment amplified from cDNA of P19 day 4 differentiated cells, comprising of the first three *Hoxd4* exons into pCR4-TOPO vector using the TOPO® TA Cloning® Kit (Invitrogen).

The non IRES Reporter plasmid was made by cloning a 3.2 kb fragment amplified from PSNlacZpA (Zhang *et al.* 2000) starting from the *Hoxd4* ATG start codon and extending throughout the entire *lacZ* coding sequence into SpeI and PmeI sites in the pMIR-REPORT Luciferase plasmid from the PSNlacZpA (Zhang et al. 2000) starting from approximately 140 bp upstream from the Drosha cleavage site of *miR-10b* to the sequence just 5′ of the *Hoxd4* ATG start codon into SpeI sites in the non IRES Reporter plasmid.

The *Hoxd4* P1 plasmid used for RNA FISH was made by cloning a 1.5 kb PCR fragment from the P1 Drosha cleavage site extending to the end of Hoxd4 exon 5 (Fig 4A, Hoxd4 P1-Cy3) into pCR4-TOPO T-tailed vector (Invitrogen). Primer sequences: P1-start(4255)-F (tatggtcgatgcaaaaacttca), Hoxd4 ex5(5802)-R (atccaagggtagaccacagc).
